# Twelve-Year Follow-Up of Laser In Situ Keratomileusis for Moderate to High Myopia

**DOI:** 10.1155/2017/9391436

**Published:** 2017-05-17

**Authors:** Tetsuya Ikeda, Kimiya Shimizu, Akihito Igarashi, Sumie Kasahara, Kazutaka Kamiya

**Affiliations:** Department of Ophthalmology, University of Kitasato School of Medicine, Kanagawa, Japan

## Abstract

**Purpose:**

To assess the long-term clinical outcomes of conventional laser in situ keratomileusis (LASIK) for moderate to high myopia.

**Methods:**

We retrospectively examined sixty-eight eyes of 37 consecutive patients who underwent conventional LASIK for the correction of myopia (−3.00 to −12.75 diopters (D)). At 3 months and 1, 4, 8, and 12 years postoperatively, we assessed the safety, efficacy, predictability, stability, mean keratometry, central corneal thickness, and adverse events.

**Results:**

The safety and efficacy indices were 0.82 ± 0.29 and 0.67 ± 0.37, respectively, 12 years postoperatively. At 12 years, 53% and 75% of the eyes were within 0.5 and 1.0 D, respectively, of the targeted correction. Manifest refraction changes of −0.74 ± 0.99 D occurred from 3 months to 12 years after LASIK (*p* < 0.001). We found a significant correlation of refractive regression with the changes in keratometric readings from 3 months to 12 years postoperatively (Pearson correlation coefficient, *r* = −0.28, *p* = 0.02), but not with the changes in central corneal thickness (*r* = −0.08, *p* = 0.63). No vision-threatening complications occurred in any case.

**Conclusions:**

Conventional LASIK offered good safety outcomes during the 12-year observation period. However, the efficacy and the predictability gradually decreased with time owing to myopic regression in relation to corneal steepening.

## 1. Introduction

Since laser in situ keratomileusis (LASIK) has been proposed [1], it has been widely accepted as a useful refractive surgical technique noted for effective outcomes in the correction of refractive errors [2–9]. However, a large amount of excimer laser photoablation may result in the deterioration of superior intrinsic corneal optical characteristic [10]. Until now, several previous studies on the long-term outcomes of LASIK have been published [11–20]. Considering that LASIK requires the excimer laser photoablation as well as the creation of the corneal flap, it is possible that corneal structural change gradually occurs in the long term as a result of corneal biomechanical weakening and that it induces a myopic shift and subsequent deterioration of visual performance [21]. In view of the prevalence of this surgery, more long-term studies among different groups are required for confirming the authenticity of these results. The aim of the current study is to retrospectively evaluate the long-term (12-year) safety, efficacy, predictability, and stability of LASIK for the correction of myopia and myopic astigmatism, with special attention to the analysis of refractive regression after this surgery.

## 2. Materials and Methods

### 2.1. Study Population

Sixty-eight eyes of the 37 consecutive patients (14 men and 23 women) who underwent conventional LASIK for the correction of myopia and myopic astigmatism, and who regularly returned for postoperative examination, as well as completing a 12-year follow-up, were included in this retrospective study. The sample size in the study offered 90.1% statistical power at the 5% level in order to detect a 0.10 difference in the logarithm of the minimal angle of resolution (logMAR) of visual acuity, when the standard deviation (SD) of the mean difference was 0.25, and offered 98.1% statistical power at the 5% level in order to detect a 0.5 D-difference, when the SD of the mean difference was 1.0 D. Eligible patients met the following inclusion criteria: unsatisfactory correction with spectacles or contact lenses, sufficient corneal thickness (estimated total corneal thickness ≥ 400 *μ*m, and anticipated residual thickness of the stromal bed ≥ 250 *μ*m after laser ablation), endothelial cell density ≥ 1800 cell/mm^2^, no history of ocular surgery, severe dry eye, progressive corneal degeneration, cataract, glaucoma, or uveitis. Keratoconic eyes were excluded by the use of the keratoconus screening test of Placido disk videokeratography (TMS-2, Tomey, Nagoya, Japan). Eyes that underwent additional LASIK enhancement surgery were also excluded from the study. Written informed consent for the LASIK surgery was obtained from all patients after explanation of the nature and possible consequences of the study. The retrospective review of the data was approved by the Institutional Review Board of Kitasato University and followed the tenets of the Declaration of Helsinki. The author's Institutional Review Board waived the requirement for informed consent for this retrospective study.

### 2.2. Surgical Procedure

After topical anesthesia, the LSK-1 microkeratome (Moria, Antony, France) was utilized to make a nasally hinged corneal flap of 130-*μ*m thickness. LASIK was performed with the VISX STAR S2 excimer laser system™ (Abbott Medical Optics, Inc, USA) to apply a broad-beam profile and an active eye tracker using an average fluency of 160 mJ/cm^2^ with a repetition rate of 10 Hz. We used a 6-mm optical zone for LASIK in this study. In all eyes, the preoperative manifest refraction was selected as the target myopic correction and we did not utilize any special nomogram in the present study. Antibiotic (0.5% levofloxacin, Cravit™, Santen, Japan) and steroidal (0.1% fluorometholone, Flumetholone™, Santen, Japan) medications were topically administered 4 times daily for 2 weeks postoperatively

### 2.3. Clinical Evaluation

Before surgery, and 3 months and 1, 4, 8, and 12 years after surgery, we assessed the following parameters: logMAR of uncorrected distance visual acuity (UDVA), logMAR of corrected distance visual acuity (CDVA), manifest refraction (spherical equivalent), intraocular pressure (IOP), and endothelial cell density, and keratometric readings, in addition to the usual slit-lamp biomicroscopic and funduscopic examinations. The safety index was determined as mean postoperative decimal CDVA/mean preoperative CDVA and the efficacy index as mean postoperative decimal UDVA/mean preoperative CDVA. Preoperatively, we measured the mean keratometric readings using an autorefractometer (ARK-700A, Nidek, Gamagori, Japan) and the central corneal thickness using an ultrasound pachymeter (DGH-500, DGH Technologies, Exton, US), the IOP using a noncontact tonometer (KT-500, Kowa, Tokyo, Japan), and the endothelial cell density using a noncontact specular microscope (SP-8800, Konan, Nishinomiya, Japan). Experienced optometrists performed at least 3 consecutive measurements in all subjects, and we used the average value for statistical analysis.

### 2.4. Statistical Analysis

All statistical analyses were performed using a commercially available statistical software (Excel-Toukei 2010, Social Survey Research Information Co, Ltd., Tokyo, Japan). One-way analysis of variance (ANOVA) was used for the analysis of the time course of changes, the Dunnett test being employed for multiple comparisons. The normality of all data samples was checked by the Kolmogorov-Smirnov test. Because the use of parametric statistics was possible, the paired *t*-test was used to compare the pre- and post-LASIK data, and the Pearson correlation coefficient was used to evaluate the correlation of myopic regression with the changes in central corneal thickness or mean keratometric readings. The results are expressed as mean ± SD, and a value of *p* < 0.05 was considered statistically significant.

## 3. Results

### 3.1. Study Population

Table 1 shows the preoperative demographics of the study population. Based on the degree of myopia, we divided them into the two groups; Group 1 (low to moderate myopia group, 30 eyes; manifest spherical equivalent < −6 D) and Group 2 (high myopia group, 38 eyes; manifest spherical equivalent ≥ −6 D). All surgeries were uneventful and no significant intraoperative complication was found.

### 3.2. Safety Outcomes

**Figure 1:**
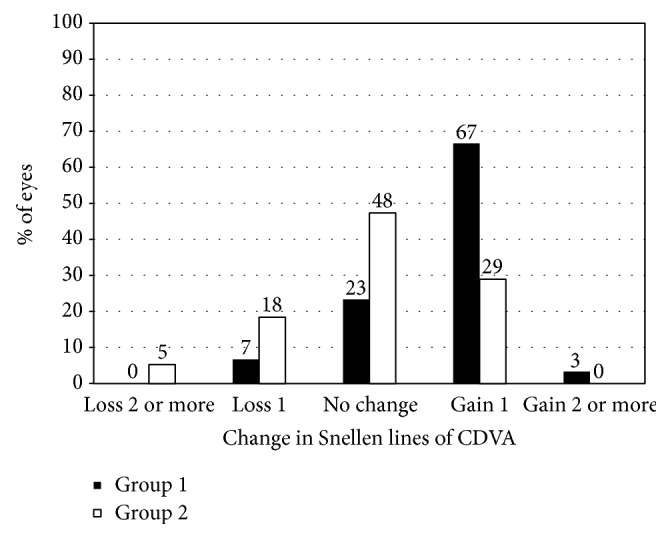
Changes in corrected distance visual acuity (CDVA) 12 years after laser in situ keratomileusis (LASIK). Group 1 is low to moderate myopia group and Group 2 is high myopia group.

LogMAR CDVA was −0.09 ± 0.08, −0.12 ± 0.09, −0.14 ± 0.09, −0.15 ± 0.10, and −0.13 ± 0.10, 3 months and 1, 4, 8, and 12 years after surgery, respectively. The safety indexes were 1.01 ± 0.17, 1.07 ± 0.17, 1.12 ± 0.24, 1.15 ± 0.24, and 1.09 ± 0.21, 3 months and 1, 4, 8, and 12 years after surgery, respectively.  Figure 1 shows changes in CDVA 12 years after LASIK.

### 3.3. Effectiveness Outcomes

**Figure 2:**
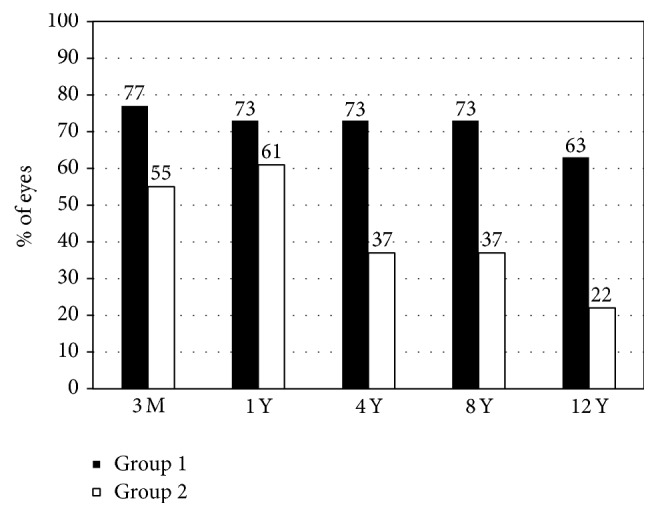
Cumulative percentages of eyes attaining uncorrected distance visual acuity (UDVA) of 20/20 or more 12 years after laser in situ keratomileusis (LASIK). Group 1 is low to moderate myopia group and Group 2 is high myopia group.

LogMAR UDVA was 0.02 ± 0.22, 0.02 ± 0.25, 0.15 ± 0.36, 0.15 ± 0.37, and 0.18 ± 0.39, 3 months and 1, 4, 8, and 12 years after surgery, respectively. The efficacy indexes were 0.82 ± 0.29, 0.83 ± 0.28, 0.71 ± 0.38, 0.71 ± 0.38, and 0.67 ± 0.37, 3 months and 1, 4, 8, and 12 years after surgery, respectively.  Figure 2 shows cumulative percentages of eyes attaining UDVA of 20/20 or more after LASIK.

### 3.4. Predictability

**Figure 3:**
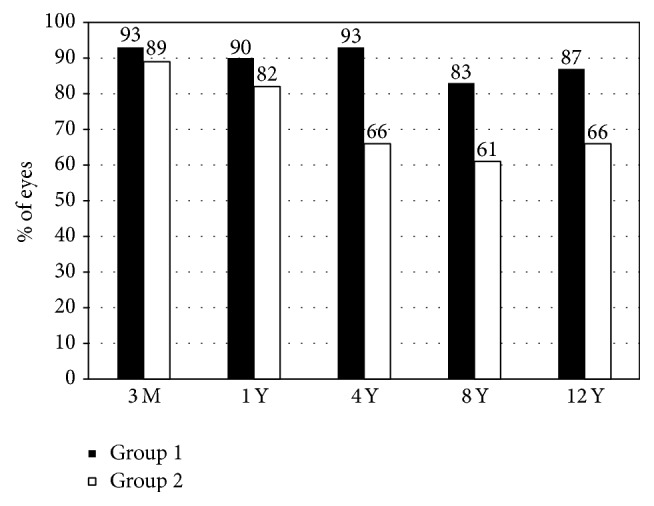
Percentages of eyes within ±1.0 D of the attempted (spherical equivalent) correction after laser in situ keratomileusis (LASIK). Group 1 is low to moderate myopia group and Group 2 is high myopia group.

**Figure 4:**
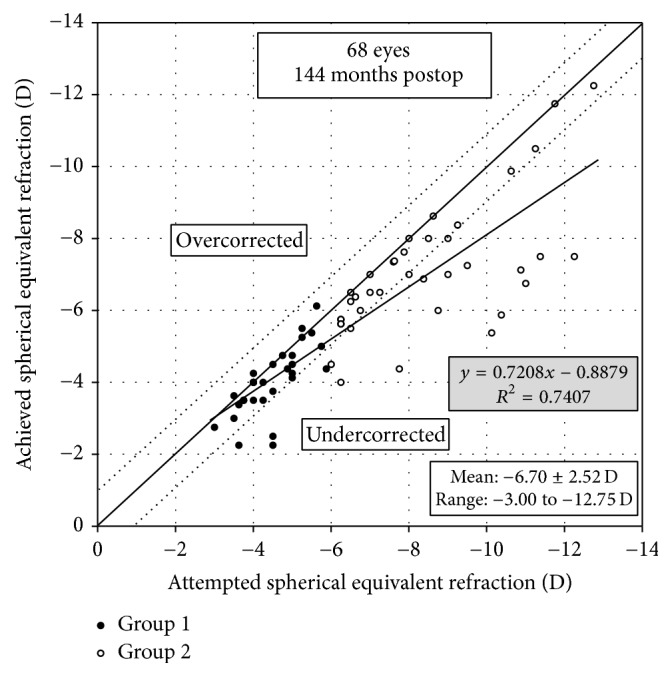
A scatter plot of the attempted versus the achieved manifest spherical equivalent correction 12 years after laser in situ keratomileusis (LASIK). Group 1 is low to moderate myopia group and Group 2 is high myopia group.

Figure 3 shows percentages of eyes within ±1.0 D of the attempted (spherical equivalent) correction after LASIK.  Figure 4 shows a scatter plot of the attempted versus the achieved correction 12 years after LASIK.

### 3.5. Stability

**Figure 5:**
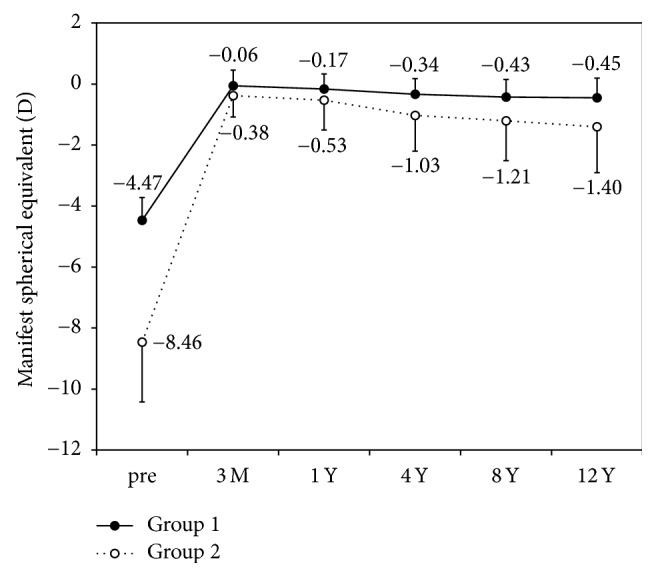
Time course of manifest spherical equivalent after laser in situ keratomileusis (LASIK). Group 1 is low to moderate myopia group and Group 2 is high myopia group.

Figure 5 shows the change in the manifest spherical equivalent. Time course changes in manifest spherical equivalent were statistically significant (ANOVA, *p* < 0.001). Multiple comparisons demonstrated significant differences between measurements made at 3 months after and at 4 years (Dunnett test, *p* = 0.02), 8 years (*p* = 0.001), and 12 years after surgery (*p* < 0.001). The changes in manifest refraction from 3 months to 1 year, from 1 year to 4 years, from 4 years to 8 years, and from 8 years to 12 years were −0.13 ± 0.51, −0.31 ± 0.65, −0.14 ± 0.50, and −0.12 ± 0.52 D, respectively.

### 3.6. Intraocular Pressure

The IOP was 15.0 ± 2.3, 10.2 ± 1.7, 10.7 ± 1.5, 11.9 ± 2.3, 11.3 ± 1.9, and 12.1 ± 2.8 mmHg, before surgery, and 3 months and 1, 4, 8, and 12 years after surgery, respectively. Time course changes in IOP were statistically significant (ANOVA, *p* < 0.001). Multiple comparisons demonstrated significant differences between measurements made before surgery and at 3 months (Dunnett test, *p* < 0.001), 1 year (*p* < 0.001), 4 years (*p* < 0.001), 8 years (*p* < 0.001), and 12 years after surgery (*p* < 0.001).

### 3.7. Endothelial Cell Density

The respective endothelial cell densities before surgery and 3 months and 1, 4, 8, and 12 years after surgery were 2725 ± 265, 2751 ± 283, 2748 ± 279, 2726 ± 335, 2671 ± 283, and 2642 ± 269 cells/mm^2^. Time course changes in endothelial cell density were not statistically significant (ANOVA, *p* = 0.16).

### 3.8. Mean Keratometry

**Figure 6:**
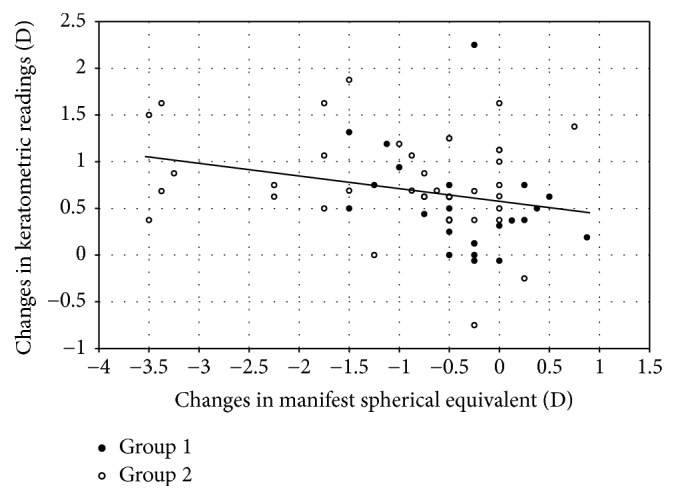
A graph showing a significant correlation between the change in mean keratometric readings and the changes in spherical equivalent from 3 month to 12 years after laser in situ keratomileusis (LASIK). Group 1 is low to moderate myopia group and Group 2 is high myopia group.

The mean keratometric readings were 38.8 ± 1.6, 38.9 ± 1.5, 39.3 ± 1.5, 39.4 ± 1.5, and 39.5 ± 1.6 D, 3 month and 1, 4, 8, and 12 years after surgery, respectively. Time course changes in mean keratometric readings were not statistically significant (ANOVA, *p* = 0.06). We found a significant correlation between the changes in mean keratometric readings and the amount of myopic regression from 3 months to 12 years after surgery (Pearson correlation coefficient, *r* = −0.28, *p* = 0.02) (Figure 6).

### 3.9. Central Corneal Thickness

The central corneal thicknesses were 480.7 ± 40.4, 484.6 ± 40.6, 485.1 ± 41.7, 488.7 ± 39.5, and 493.1 ± 42.9 *μ*m, 3 month and 1, 4, 8, and 12 years after surgery, respectively (ANOVA, *p* = 0.63). We found no significant correlation between the changes in central corneal thickness and the amount of regression from 3 months to 12 years after surgery (*r* = −0.08, *p* = 0.63).

### 3.10. Adverse Events/Secondary Surgeries

Of the 68 eyes examined, two (2.9%) developed symptomatic cataracts, and these cataracts lost 2 or more lines in CDVA. Twenty eyes (29.4%) developed mild dry eye so that artificial tears were required. Twelve eyes (17.6%) required received additional topical application of the intraocular pressure-lowering drug (2.5% nipradilol, Kowa, Tokyo, Japan) [22, 23]. Otherwise, neither epithelial ingrowth, diffuse lamellar keratitis, iatrogenic ectasia, and severe dry eye, nor any other vision-threatening complication was observed at any time during the 12-year follow-up period.

## 4. Discussion

In the present study, our results showed that LASIK offered outcomes with a high degree of safety in the correction of myopia and myopic astigmatism throughout the 12-year follow-up period. However, it should be noted that a slight decrease in efficacy and predictability was observed with time, possibly because most eyes showed some amount (approximately 10%) of myopic regression after LASIK.

It is of clinical importance to elucidate the long-term outcomes of LASIK not only for the surgeons, but also for the patients undergoing this surgery. To date, there have been several studies published over a span of more than 10 years that have examined the long-term results of this surgery (Table 2). With regard to myopic regression after LASIK, Alió et al. reviewed the 15-year clinical results of LASIK and found that the myopic regression from 3 months to 15 years postoperatively was −1.66 ± 2.15 D in eyes with myopia of −9.47 ± 3.26 D, indicating that the refractive outcomes tended to shift toward undercorrection over time [19]. In another study also, regression was seen, in this case at annual rates of −0.12 ± 0.15 D and −0.25 ± 0.18 D, in eyes with myopia of up to −10.0 D [13] and over −10.0 D [14], respectively. Kymionis et al. demonstrated that the myopic regression was −1.14 ± 1.67 D at 11 years after LASIK in eyes with myopia of −12.96 ± 3.17 D [11]. Rosman et al. stated that mean regression at 10 years after LASIK was −1.86 D in eyes with myopia of −14.33 ± 2.11 D [17]. Oruçoğlu et al. and Lim et al. described that mean final refraction was −6.09 D and −1.09 D in eyes with myopia of −21.70 ± 5.86 D and −5.73 ± 2.76 D, respectively [18, 20]. In the current study, similarly, we found refractive regression of −0.74 ± 0.99 D from 3 months to 12 years postoperatively. All these findings indicate that myopic regression does occur in most cases after LASIK, especially when the amount of myopic correction is large. We believe that this information is helpful, not only for the surgeons involved, but also for the patients, since it offers an understanding of the long-term prognosis of LASIK in a clinical setting.

Although the exact etiology still remains unclear, possible mechanisms leading to myopic regression after LASIK have been considered to include compensatory epithelial hyperplasia [24, 25], corneal forward shift [21, 26, 27], corneal hydration, stromal synthesis, nuclear sclerosis of the crystalline lens [28, 29], and axial elongation [30–32]. In this study, we found a weak but significant correlation between refractive regression and the changes occurring in keratometric readings from 3 months to 12 years postoperatively, indicating that the steepening of the cornea is one of possible sources of myopic regression after surgery. However, the patient age was 34.4 ± 9.5 years, which was relatively older than that in other studies on LASIK. As evidenced by the small *r* value, other contributing factors such as the change in nuclear sclerosis of the crystalline lens or that in axial length, possibly due to relatively old patient age and high myopic eyes, may play some role in refractive regression in this series. The post-LASIK changes in manifest spherical equivalent have been reported to show a statistically significant decrease over time, whereas the change in keratometry was stable over time, demonstrating that there was no significant association between the rate of change in keratometry and that in spherical equivalent [33].

Our study had the following limitations: firstly, it was conducted retrospectively. Since a retrospective study may include some bias such as information bias and selection bias, the evidence level is not very high in this study. Secondly, only consecutive patients who completed a 12-year follow-up were included. Considering that the patients who were satisfied with their visual performance after refractive surgery tended to be lost to the follow-up, we cannot deny the possibility that there existed some bias in patient selection in the present study. A prospective randomized, controlled study may provide further information that will confirm the validity of these results. Thirdly, we included both eyes of each patient undergoing LASIK in the current study, although only one eye per patient should be included for statistical analysis. We confirmed that similar results were obtained with LASIK, even when only one eye was chosen randomly from each patient, and we therefore enrolled both eyes of the same patient, following the descriptions of most published studies on refractive surgery. Fourthly, the sample size was relatively small in this study. We should be aware that our results cannot be generalized to the millions of patients undergoing LASIK. Fifthly, we did not evaluate the detailed visual performance after LASIK, such as higher-order aberrations, intraocular scattering, contrast sensitivity function, halos, and stereopsis, since we performed LASIK many years ago in this series. These assessments may provide further information for understanding the postoperative visual quality in depth.

In summary, our results supports the view that LASIK offered outcomes with good safety standards in the correction of myopia and myopic astigmatism throughout the 12-year observation period. It is indicated that LASIK is a safe surgical option for such patients in a clinical setting. However, we should be aware that the efficacy and the predictability of this surgical procedure decreased slightly with time during the 12-year observation period, since most eyes suffered some amount (approximately 10%) of myopic regression in association with corneal steepening after LASIK.

## Figures and Tables

**Table 1:** Preoperative demographics of the study population undergoing conventional laser in situ keratomileusis (LASIK).

Characteristic	Mean ± standard deviation
Age (years)	34.4 ± 9.5 years (range, 23 to 58 years)
Gender (% female)	63.2%
Manifest spherical equivalent (D)	−6.70 ± 2.52 D (range, −3.00 to −12.75 D)
Manifest cylinder (D)	0.76 ± 0.67 D (range, 0.00 to 3.00 D)
LogMAR UDVA	1.32 ± 0.22 (range, 0.82 to 2.00)
LogMAR CDVA	−0.10 ± 0.05 (range, −0.18 to 0.00)
Mean keratometric readings (D)	43.6 ± 1.3 D (range, 40.5 to 47.4 D)
Central cornea thickness (*μ*m)	545.4 ± 30.6 *μ*m (range, 479 to 612 *μ*m)
Intraocular pressure (mmHg)	15.0 ± 2.3 mmHg (range, 9 to 20 mmHg)
Endothelial cell density (cells/mm^2^)	2725 ± 265 cells/mm^2^ (range, 1990 to 3246 cells/mm^2^)

D = diopter, LogMAR = logarithm of the minimal angle of resolution, UDVA = uncorrected distance visual acuity, and CDVA = corrected distance visual acuity.

**Table 2:** Summary for long-term clinical outcomes of conventional laser in situ keratomileusis (LASIK).

Author Year	Excimer laser	Eyes	Follow-up (years)	Mean Age (years)	Mean spherical equivalent (D)	Safety index	Efficacy index	Predictability within ±1.0 D (%)	Myopic regression (D)	Retreatment (%)
Alió et al. 2009	VISX 20/20	34	10	29.4	−8.30	1.11	0.95	88	−0.97 3 months to 10 years	18
Alió et al. 2008	VISX 20/20	97	10	33.2	−7.27	1.08	0.88	73	−1.04 3 months to 10 years	20.6
Alió et al. 2008	VISX 20/20	196	10	32.9	−13.95	1.21	0.77	42	−1.83 3 months to 10 years	27.5
Kymionis et al. 2007	MEL 60	11	11	41.7	−12.96	N.A.	N.A.	55	−1.14	0
Rosman et al. 2011	VISX 20/20	126	10	33.7	−14.33	1.20	0.84	42	−1.86 3 months to 10 years	28.6
Oruçoğlu et al. 2012	Keracor	36	≥10	N.A.	−21.7	1.48	0.29	10 within ±2.0 D	−6.09	N.A.
Alió et al. 2015	VISX 20/20	40	15	51.1	−9.47	1.23	0.95	46	−1.66 3 months to 15 years	30.8
Lim et al. 2016	VISX S4	36	10	26.6	−5.73	0.99	0.73	68	−1.09	0 (excluded)
Current	VISX STAR S2	68	12	34.4	−6.70	1.09	0.67	75	−0.74 3 months to 12 years	17.6

D = diopter, N.A. = not applicable.
